# African Americans with a family history of cardiovascular disease show lower endothelial‐dependent vasodilation

**DOI:** 10.14814/phy2.70176

**Published:** 2025-03-28

**Authors:** Julian F. Thayer, Darcianne K. Watanabe, Julia Birenbaum, Julian Koenig, Marc Jarczok, DeWayne P. Williams, Gaston K. Kapuku

**Affiliations:** ^1^ Department of Psychological Science University of California Irvine California USA; ^2^ School of Social Ecology University of California Irvine California USA; ^3^ Faculty of Medicine and University Hospital Cologne, Department of Child and Adolescent Psychiatry, Psychosomatics and Psychotherapy University of Cologne Cologne Germany; ^4^ Department of Psychosomatic Medicine and Psychotherapy Ulm University Medical Center Ulm Germany; ^5^ Department of Pediatrics and Medicine, Georgia Prevention Institute Augusta University Augusta Georgia USA

**Keywords:** endothelium, health disparities, heart rate variability, prehypertension, total peripheral resistance

## Abstract

Normotensive African Americans (AAs) show attenuated vascular responses and reduced nitric oxide (NO) bioavailability compared to European Americans (EAs). Few studies have used diverse measures to examine differences in macrovascular function and structure in individuals with a family history of CV disease (CVD). We assessed 150 AAs (M_age_, 23.57 ± 2.73 yr) and 104 EAs (Mage, 22.70 ± 2.86) with a confirmed family history of CVD. Age, sex, body mass index, and father's education were used as covariates, hemodynamic measures (heart‐rate [HR], stroke volume [SV], cardiac output [CO], total peripheral resistance [TPR], mean arterial pressure [MAP], systolic and diastolic blood pressure [SBP/DBP], and pulse pressure [PP]), high‐frequency heart‐rate variability [HF‐HRV], and endothelial‐dependent arterial dilation [EDAD] were the dependent variables. AA's had lower EDAD (11.64 vs. 13.20%) and higher HF‐HRV (7.31 vs. 7.11 ms^2^), TPR (17.60 vs. 15.93 mmHg/L/min), TPI (33.72 vs. 30.09 mmHg/L/min/m^2^), MAP (83.60 vs. 78.36 mmHg), SBP (115.44 vs. 110.23 mmHg), and DBP (65.35 vs. 60.57 mmHg). Lower EDAD alongside no ethnic differences in PP, HR, or SV suggests early onset endothelial dysfunction (lower NO availability) rather than inherited pathophysiological structural characteristics (arterial stiffness) in AAs. Future prospective studies are needed and should consider measures of sympathetic activity and potential moderators, including discrimination.

## 1 INTRODUCTION

Hypertension is a leading cause of cardiovascular morbidity and mortality. However, African Americans (AAs) have been shown to have a greater risk of poor cardiovascular outcomes compared to similar European Americans (EAs), and signs of hypertension‐related differences appear earlier in AAs than in EAs (Tsao et al., 2023). Whereas vascular factors have long been implicated in this health disparity (Anderson, 1989; Taherzadeh et al., 2010), additional research is needed to further explicate the precise aspects of vascular structure and function that may contribute to this persistent disparity. In a comprehensive review of structural and functional differences in resistance vessels between AAs and EAs, several important conclusions were drawn (Taherzadeh et al., 2010). First, compared to normotensive EAs, normotensive AAs have attenuated vasodilatory responses. Second, reduced nitric oxide (NO) bioavailability appears to be the most important factor distinguishing AA from EA vessels. Finally, the available data do not provide evidence for elevated sympathetic nervous system activity in AAs.

Thus, the present study sought to further elaborate on the vascular differences between AAs and EAs. First, we sought to examine indices of macrovascular function (i.e., large, conduit arteries [e.g., brachial, radial]) and structure in a sample of emerging adults at risk for cardiovascular disease (CVD) because of a confirmed family history of CVD (G. K. Kapuku et al., 2002, 2004; Sandoo et al., 2010; Su et al., 2014). Individuals with a family history of CVD are known to be at increased risk for CVD. However, despite the link between CVD and vascular function (Brunner et al., 2005; Taddei & Salvetti, 2002), to date, few studies have examined vascular function in such individuals while simultaneously using a diverse range of measures. Moreover, pulse pressure (PP) is a function of ventricular ejection and arterial stiffness and has been shown to be an independent predictor of CVD risk (Laurent et al., 2006; Mackenzie et al., 2002; Mitchell, 2009; Van Bortel et al., 2001). Thus, our second aim was to determine if the observed differences in vascular function in this sample of normotensive emerging adults with a family history of CVD were driven by structural or functional vascular factors at this early age. Finally, hypertension characterized by elevated total peripheral resistance (TPR), a measure of resistance to blood flow in the vasculature (Brook & Julius, 2000; Guyton & Hall, 2000), rather than CO has been linked with an increased risk of CV events, morbidity, and mortality (Fagard et al., 1996; Mensah et al., 1993). In EAs, higher parasympathetic activity, indexed by heart‐rate variability (HRV), the beat‐to‐beat variability between successive heartbeats, has been shown to predict lower TPR 6 years later. However, in AAs, this relationship was greatly attenuated (Williams et al., 2021). Additionally, higher vagal activity should be associated with lower blood pressure and, specifically, TPR. However, a pattern of greater HRV and greater TPR in AAs and other individuals exposed to discrimination has been identified by our group and termed the cardiovascular conundrum (Hill et al., 2021; Williams et al., 2021). Thus, our third aim was to examine differences in TPR and HRV. We hypothesized that AA emerging adults with a family history of CVD would show greater TPR and HRV relative to their EA counterparts. We also hypothesized that AAs would show signs of reduced endothelial‐dependent macrovascular function as indexed by reactive hyperemia‐induced endothelial‐dependent arterial dilation (EDAD). Finally, by examining PP, we could investigate if the differences in vascular function between AAs and EAs were due to early or inherited structural vascular differences.

## 2 METHODS

### 2.1 Subjects

The present study used data from the Augusta Heart Study (AHS)—a longitudinal study investigating the development of cardiovascular health and risk in young, healthy, normotensive adults with a verified family history of CVD (i.e., hypertension and myocardial infarction). If at least one biological parent and one grandparent had essential hypertension, and this was verified by the individual's personal physicians, we considered the individual as having a verified family history of CVD (G. Kapuku et al., 2019; Treiber et al., 1994) (Kapuku et al., 2019; Treiber et al., 1994). Participants were instructed to refrain from smoking 5 h prior to the visit and alcohol and illicit drugs 6 h prior. Self‐reports indicated that all complied. Participants also reported that they were not taking vasoactive medications. When possible, assessments were conducted while participants were fasting. Recruitment methods for the AHS have been previously reported (Hao et al., 2021; G. K. Kapuku et al., 2004; Williams et al., 2021), and all procedures were approved by the Medical College of Georgia's Human Assurance Committee. All participants provided written informed consent, and data were available for all variables of interest in the full sample (*N* = 295), including 163 women, 132 men, 152 African American (AA*s*) individuals, and 103 European American individuals (EA*s*). Any exclusion of subjects from the original AHS dataset (*N* = 4) was due to a lack of or missing data on the variables of interest.

### 2.2 Demographic and anthropometric measures

Demographic variables collected at baseline included sex assigned at birth (female/male), ethnicity (parent/guardian reported as AA or EA), height (meters; m), weight (kilograms; kg), age (in years), and father's education (in years). Sex, ethnicity, age, and education were reported by participants' parent(s) or guardian(s). Height and weight were collected using a calibrated Health‐O‐Meter medical scale. Body mass index (BMI) was calculated as weight divided by height squared (weight/height^2^; kg/m^2^).

### 2.3 Hemodynamic assessment

Heart rate (HR) and blood pressure measurements (systolic blood pressure [SBP] and diastolic blood pressure [DBP]) were obtained using a Dinamap model 1846 SX oscillometric BP device (Critikon, Inc., Tampa, FL), while participants were in the supine position. Pulse pressure (PP) was computed as the difference between SBP and DBP and is associated with both structural and functional aspects of the vasculature (Van Bortel et al., 2001). Mean arterial pressure (MAP) was computed by averaging three blood pressure readings recorded during the baseline visit. Cardiac output (CO) and stroke volume (SV) were measured using bioimpedance cardiography (NCCOM‐3, BoMeD Medical Manufacturing, Ltd., Big Lake, MN). TPR was calculated by dividing MAP by CO. Lastly, the total peripheral resistance index (TPI), as well as the cardiac index (CI), were computed by adjusting for body surface area (0.7184*weight, kg^0.425^ *height, m^20.725^) (G. K. Kapuku et al., 2004; Sherwood et al., 1990; Zhu et al., 2007). For the primary analyses, CI and TPI were the main indicators of CO and TPR, respectively. Pulse pressure (PP) is a readily available marker of arterial stiffness, can be derived from clinical BP readings, and is widely used as an indirect surrogate marker of arterial stiffness (Mackenzie et al., 2002). As such, PP was computed as mean SBP−mean DBP and included as a measure of both vascular structure and function (Laurent et al., 2006; Muntner et al., 2019; Van Bortel et al., 2001) (Laurent et al., 2006; Muntner et al., 2019; Van Bortel et al., 2001).

### 2.4 Electrocardiographic assessment

Heart rate variability (HRV) was collected using a short‐term (5 min) resting electrocardiogram (ECG) recording. The ECG measures the heart's electrical activity, with peaks (R‐spikes) signifying ventricular contractions, and the time between R‐spikes, or R‐R intervals (ms), representing the duration between heartbeats (inter‐beat‐intervals). R‐R interval data were filtered based on two criteria: (1) R‐R intervals ranging between 300 and 2000 ms, and (2) consecutive R‐R interval ratios between 0.8 and 1.2. This filtering ensured artifacts were excluded, and only physiologically relevant data remained. Filtered R‐R intervals were then analyzed using Kubios HRV software (Tarvainen et al., 2013).

This study included the high‐frequency (HF) power (0.15–0.40 Hz) component of HRV (HF‐HRV). HF‐HRV was calculated using Fast Fourier Transformation and expressed in milliseconds squared (ms^2^). To address skewness, HF‐HRV was then log‐transformed using the natural logarithm. HF‐HRV is considered a reliable indicator of parasympathetic (vagal) cardiac control and has been extensively used in cardiovascular research (Hillebrand et al., 2013; Laborde et al., 2017; Shaffer & Ginsberg, 2017; Task Force of the European Society of Cardiology & The North American Society of Pacing and Electrophysiology, 1996).

### 2.5 Endothelial‐dependent arterial dilation assessment

To test our hypothesis that AAs would show signs of reduced endothelial‐dependent macrovascular function, the technique proposed by Celermajer et al. (1992) was used to index reactive hyperemia‐induced EDAD (Joannides et al., 1995; Kooijman et al., 2008; Lieberman et al., 1996; Mullen et al., 2001; Rubanyi et al., 1986; Sandoo et al., 2010). Brachial artery (BA) images were obtained using a Hewlett‐Packard Sonos echocardiograph (Andover, MA) with a 7.5 MHz linear array transducer in sector mode. Ultrasound images of the BA were captured before and after induced reactive hyperemia, which involved occluding the BA for four (4) minutes with a BP pressure cuff inflated to 220–240 mmHg (Celermajer et al., 1992). Preocclusion and postocclusion two‐dimensional BA images were recorded for 30 s and continuously for two (2) minutes after release of the BP cuff, respectively. All images were stored on VHS tapes.

The BA walls at the “m” line (i.e., intima‐media junction) were automatically identified, and arterial diameters were derived using Brachial Tool software (Medical Imaging Application, Iowa Cita, IA). Ten frames from the preocclusion period and 120 from the postocclusion period were analyzed. The post‐occlusion frame with the greatest diameter was used to calculate maximum dilation. Our laboratory has previously reported that this method is highly correlated with the polynomial curve fitting method (*r* = 0.90) (G. K. Kapuku et al., 2004). EDAD was computed as the percent change in BA diameter between the average preocclusion BA diameter and maximum postocclusion BA diameter (Celermajer et al., 1992). Preocclusion and postocclusion measured diameters by two observers showed high reliability for both measures (*r* = 0.99 and 0.98, respectively).

### 2.6 Statistical analysis

All statistical tests were conducted using SPSS (v. 28, IBM, Armonk, NY, USA). Two‐tailed tests were conducted for all variables except HF‐HRV, TPR, and EDAD, which reflect one‐tailed tests corresponding to our directional hypotheses. Significance levels were evaluated using an alpha of 0.05. We first stratified subjects into groups based on their reported ethnicities (AAs and EAs). Chi‐square tests of ethnic differences in sex and *t*‐tests of ethnic differences in covariates age (in years), body mass index (BMI), and education were conducted. T‐statistics (*t*), effect sizes (*r*), associated 95% confidence intervals, and *p*‐values are reported.

To test our hypothesis that AA emerging adults with a family history of CVD would show greater TPR and HRV relative to their EA counterparts Analysis of Variance (ANOVA) tests were conducted to examine differences in independent variables (HF‐HRV, HR, SV, CO, CI, TPR, TPI, MAP, SBP, DBP, and PP) between ethnic groups. All differences statistically controlled for age, sex (dummy coded), education, and BMI; results were identical when considering these covariates, and thus, tests without covariates are reported. Preplanned contrasts (*t*‐tests) were used to determine differences between groups (Rosnow & Rosenthal, 2009). Extreme scores were evaluated, and three participants were removed as they had studentized residual scores that exceeded the threshold of an absolute value of 2.5 (Belsley et al., 1980). Statistical assumptions were met for all tests.

## 3 RESULTS

### 3.1 Sample baseline characteristics

The average age for the total sample was 23.15 [2.82] years old, with AAs average age of 23.57 (2.73) and EAs average age of 22.70 (2.86) years old. Means and standard deviations for all variables in the full sample are presented in Table 1a, and ethnicity‐stratified values are in Table 1b,c. Compared to EAs, AAs were significantly older (23.53 vs. 22.70 years old, *p =* 0.011), had significantly higher BMI (29.70 vs. 27.09 kg/m^2^, *p =* 0.005) and lower education (12.95 vs. 14.06 years, *p <* 0.001). There were significantly more AA women and EA men (*X* ^*2*^ = 5.50, *p =* 0.019). Women had significantly higher HR, TPR, and DBP (each *p <* 0.031) than men, whereas men had higher SV, CO, MAP, SBP, and PP than women (each *p <* 0.006; see Table S1).

**TABLE 1:** Descriptive statistics.

	a: Full sample		b: African Americans		c: European Americans		*t*/*X* ^2^	*r*	95% CI	*p*
	Mean	SD	Mean	SD	Mean	SD				
Age	23.15	2.82	23.53	2.69	22.70	2.86	2.55	0.15	0.03, 0.26	**0.011**
BMI, kg/m^2^	28.42	7.97	29.70	8.78	27.09	6.86	2.83	0.16	0.05, 0.27	**0.005**
Education, years	13.49	2.36	12.95	2.13	14.06	2.47	−4.15	0.24	0.12, 0.34	**<0.001**
Sex, women/men	164/132		95/58		69/74		5.50			**0.019**
HF‐HRV, ms^2^	7.21	1.03	7.31	0.94	7.11	1.12	2.00	0.12	0.00, 0.23	**0.023**
HR, bpm	64.05	9.05	64.69	9.33	63.37	8.73	0.10	0.01	−0.11, 0.12	0.922
SV, mL/beat	80.87	19.26	78.96	19.07	82.90	19.33	1.83	0.11	−0.01, 0.22	0.068
Cardiac output, L/min	5.14	1.29	5.07	1.27	5.22	1.31	1.73	0.10	−0.01, 0.21	0.085
Cardiac index, L/min/m^2^	2.65	0.57	2.61	0.60	2.70	0.53	1.43	0.08	−0.03, 0.20	0.154
TPR, mmHg/L/min	16.79	4.57	17.60	4.92	15.93	4.02	3.74	0.22	0.10, 0.32	**<0.001**
TPI, mmHg/L/min/m^2^	31.96	7.75	33.72	8.56	30.09	6.28	4.11	0.24	0.12, 0.34	**<0.001**
MAP, mmHg	81.06	8.22	83.60	8.50	78.36	6.98	5.86	0.33	0.21, 0.42	**<0.001**
SBP, mmHg	112.92	11.85	115.44	11.80	110.23	11.34	4.42	0.25	0.14, 0.35	**<0.001**
DBP, mmHg	63.03	7.77	65.35	8.21	60.57	6.43	5.22	0.29	0.18, 0.39	**<0.001**
Pulse pressure, mmHg	49.89	10.50	50.10	10.29	49.66	10.75	1.15	0.07	−0.05, 0.18	0.250
EDAD, %	12.40	7.15	11.64	6.62	13.20	7.61	2.02	0.12	0.00, 0.23	**0.022**

*Note*: This table presents the means and standard deviations of variables of interest in the full sample (a) and stratified by African Americans (b) and European Americans (c). Pearson chi‐square for differences between women and men (*X* ^2^), Means (M), standard deviations (SD), *t*‐values (*t*), effect size (*r*), associated 95% confidence intervals for *r*'s (95% CI) and analysis of variance *t*‐ and *p*‐value statistics for the difference between African Americans and European Americans. Age in years, body mass index (BMI) measured in kg/m^2^; father's education measured in years; HR (heart rate) in beats per minute; high‐frequency power heart rate variability (HF‐HRV) in milliseconds squared (ms^2^); stroke volume (SV) in milliliters per beat (mL/beat); cardiac output in liters per minute (L/min); adjusted CO (cardiac index; CI) in liters per min per meters squared (L/min/m^2^); total peripheral resistance (TPR) in millimeters of mercury per liter per minute (mmHg/L/min); body surface area adjusted TPR (TPI) in millimeters of mercury per liter per meters squared (mmHg/L/min/m^2^); mean arterial pressure (MAP) in millimeters of mercury (mmHg); systolic blood pressure (SBP) in millimeters of mercury (mmHg); diastolic blood pressure (SBP) in millimeters of mercury (mmHg); pulse pressure in millimeters of mercury (mmHg); and endothelial‐dependent arterial dilation (EDAD) expressed as a percent (%). *P*‐values for HF‐HRV, TPR, and EDAD reflect one‐tailed tests corresponding to directional hypotheses. Significant *p*‐values are bolded (*p <* 0.05).

### 3.2 Ethnic differences in all cardiovascular variables

**FIGURE 1:**
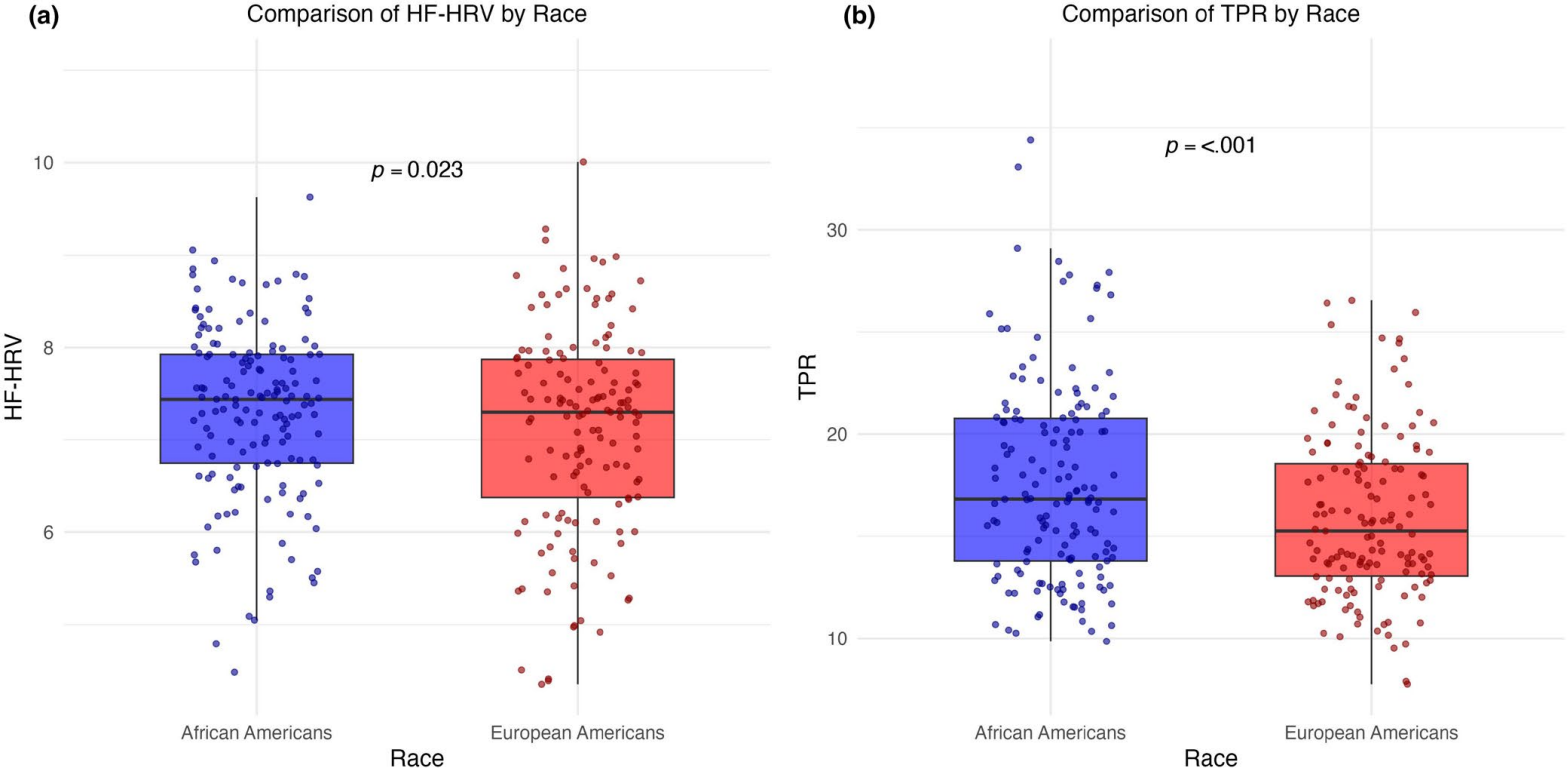
(a) and (b) depict mean differences between African Americans and European Americans in natural log‐transformed high‐frequency power heart‐rate variability in milliseconds squared (HF‐HRV) and total peripheral resistance (TPR) in millimeters of mercury per liter per minute (mmHg/l/min), respectively, with standard error bars.

**FIGURE 2:**
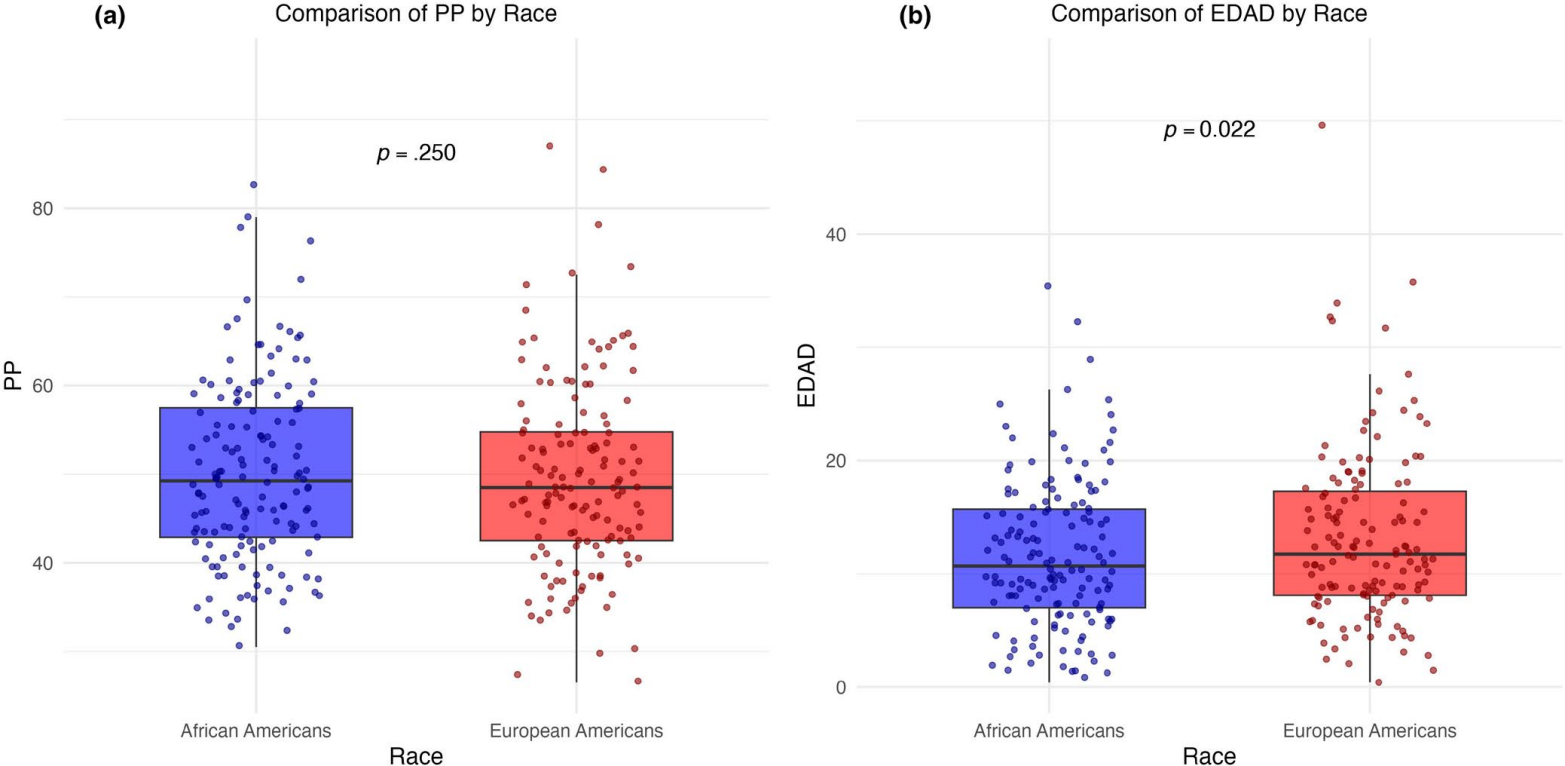
(a) and (b) depict mean differences between African Americans and European Americans in pulse pressure (PP) in millimeters of mercury (mmHg) and endothelial‐dependent arterial dilation (EDAD) expressed as a percent (%), respectively, with standard error bars.

Univariate analyses showed that AAs had significantly higher HF‐HRV (The results were substantively the same using the root mean square of successive differences between R‐R intervals (RMSSD) vs. HF‐HRV) (7.31 vs. 7.11 ms^2^, *r = 0*.12, *p =* 0.023; Figure 1), TPR (17.60 vs. 15.93 mmHg/L/min, *r = 0*.22, *p* < 0.001; Figure 1b), TPI (33.72 vs. 30.09 mmHg/L/min/m^2^, *r =* 0.24, *p* = <0.001), MAP (83.60 vs. 78.36 mmHg, *r = 0*.33, *p* = <0.001), SBP (115.44 vs. 110.23 mmHg, *r =* 0.25, *p* = <0.001), and DBP (65.35 vs. 60.57 mmHg, *r =* 0.29, *p* = <0.001) and lower EDAD (11.64 vs. 13.20%, *r = 0*.12, *p* = 0.022; Figure 2). The lack of association between race and PP remained when controlling for stroke volume (*F* [286] = 1.43, *p* = 0.233), which did not significantly predict PP (*F* [286] = 0.23, *p* = 0.630). Moreover, race was a stronger predictor of EDAD (*F* [286] = 4.069, p = 0.022) than BMI (*F* [286] = 2.66, *p* = 0.104). Differences in FMD were robust to adjustment for differences in blood pressure such that controlling for MAP, which was not a significant predictor of FMD (*F* [286] = 0.40, *p = 0*.527), race effects remained significant (*F* [286] = 2.88, *p = 0*.045). Regardless of race, EDAD was not associated with HRV both before and after adjustment (AAs: *r* = −0.02, *p* = 0.39; EAs: *r* = 0.10, *p* = 0.13), or with TPR (AAs: *r* = 0.01, *p* = 0.45; EAs: *r* = 0.06, *p* = 0.26). Finally, there were no significant differences between AAs and EAs in PP (Figure 2b), HR, SV, CO, or CI (each *r* < 0.07, *p >* 0.068).

## 4 DISCUSSION

The results of the present study in a sample of normotensive, emerging adults with a confirmed family history of CVD showed that AAs had greater TPR and lower EDAD compared to their EA counterparts. In addition, whereas AAs had greater HRV, there was no difference in PP or SV compared to their EA counterparts. The findings of higher TPR and higher HRV in AAs compared to EAs replicate our previously identified cardiovascular conundrum pattern. The present results extend these prior findings to measures of endothelial‐dependent vasodilation, indexed by EDAD, and arterial stiffness, indexed by PP, and provide evidence that the previously hypothesized mechanism for the cardiovascular conundrum pattern may indeed be decreased nitric oxide availability. In light of prior evidence, these findings suggest the possibility of earlier onset vascular dysfunction among AAs rather than the presence of inherited pathophysiological structural vascular characteristics, such as arterial stiffness, in young adults with a confirmed family history of CVD.

Flow‐mediated dilation (FMD) is thought to be a surrogate marker of macrovascular endothelial function and NO availability (Boo et al., 2002; Bramwell & Hill, 1922; Celermajer, 1997; Sandoo et al., 2010). In particular, FMD can be substantially attenuated by NO blockade, suggesting that FMD is both endothelium‐dependent and a good surrogate NO‐specific index of endothelial function (Joannides et al., 1995; Kooijman et al., 2008; Lieberman et al., 1996; Mullen et al., 2001). As such, the lower value of EDAD in AAs suggests that AAs have lower functional macrovascular activity, which has implications for risk of cardiovascular events. In particular, meta‐analyses have found that a 1% decrease in FMD was associated with a 9% increase in future risk of cardiovascular events (Green et al., 2011; Inaba et al., 2010). Moreover, an independent risk factor for CVD is PP, the major determinants of which are ventricular ejection and arterial stiffness, reflecting both structural and functional aspects of the vasculature (Laurent et al., 2006; Mitchell, 2009; Van Bortel et al., 2001). Thus, the lack of significant ethnic differences in PP, HR, and SV, and the fact that the results remained after controlling for MAP, suggests that AAs lower NO availability (i.e., FMD), found in the present study and prior work, may indicate vascular dysfunction rather than the presence of inherited pathophysiological vascular structure in AAs (Campia et al., 2002; Kappus et al., 2017; Yang et al., 2019). Although speculative, taken together, our results suggest that in AAs with a family history of CVD, the earlier onset of hypertension may manifest first as vascular dysfunction (i.e., higher TPR and reduced FMD) resulting from reduced NO availability rather than inherited pathophysiological structural vascular characteristics, such as arterial stiffness (Campia et al., 2002; Kappus et al., 2017; G. Kapuku et al., 2019; Lackland, 2007, 2009, 2014; Muntner et al., 2004; Yang et al., 2019). As a consequence of these functional changes, structural changes associated with arterial stiffness and increased end‐organ damage, including left ventricular hypertrophy, may follow, putting AAs at greater risk of hypertension‐related morbidity and mortality.

In addition, the higher values of TPR in the AAs further suggest relatively lower vasodilation in AAs. This lower vasodilation, which is associated with an endogenous vasodilatory stimulus, is consistent with prior research showing reduced vasodilation to an exogenous vasodilatory stimulus (Jones et al., 1999). For example, we recently showed that pregnant AA women, despite having lower MAP as expected in pregnancy, still had higher TPR than their EA counterparts (Christian et al., 2021). Importantly, this greater TPR in AA women was associated with discrimination such that those AA mothers who reported fewer incidences of discrimination had higher TPR. This finding was replicated in a sample of lesbian, gay, and bisexual (LGB) individuals such that those LGB individuals who reported fewer incidences of discrimination showed higher TPR (Rosati et al., 2021). The finding that a lack of reports of discrimination is associated with higher BP is not new and has been suggested to be associated with internalized racism, among other things (Krieger & Sidney, 1996). Thus, future studies should include potential moderators of both macrovascular function and TPR, such as discrimination.

Moreover, prior work has found differences between AA and EA in the underlying hemodynamics of BP and a conundrum pattern of physiological responding (Brownlow et al., 2020; Hill et al., 2015; Williams et al., 2024). Both short and long‐term BP regulation is now thought to involve the baroreflex (Lohmeier & Iliescu, 2015). Consistent with the present study, we recently reported that the vascular limb of the baroreflex was less effective in AAs compared to EAs, as indexed by the proportion of reflex changes in TPR in response to changes in SBP (Williams et al., 2024). Similarly, we have reported that whereas higher HRV predicted lower TPR 6 years later in EAs, this relationship was absent in AAs, such that higher HRV was not associated with lower TPR (Williams et al., 2021). The FMD and HRV associations in AAs and EAs in the present study were similar in magnitude to the HRV and TPR association in our prior work (Williams et al., 2021). Importantly, we have shown in meta‐analyses that AAs have higher HRV and higher TPR than their EA counterparts (Brownlow et al., 2020; Hill et al., 2015) and in experimental work that AAs show less vascular baroreflex effectiveness (Williams et al., 2021). We have postulated that this conundrum pattern of lower vascular responsiveness may be due to decreased NO availability. Although causal inferences cannot be drawn due to the cross‐sectional nature of this study, the present results provide additional evidence that reduced NO availability in AAs may be one mechanism for lower vascular responsiveness (Campia et al., 2002; Kappus et al., 2017; Williams et al., 2024; Yang et al., 2019). One potential experimental therapy shown to mitigate NO‐mediated vascular dysfunction in AAs is vitamin D supplementation (Wolf et al., 2020). However, future studies are needed to provide further insights into potential interventions to improve macrovascular function.

This study is not without limitations. First, our finding of no sex differences in EDAD conflicts with previous research showing sex differences in several measures used in the present study, including EDAD (G. K. Kapuku et al., 2004; Koenig & Thayer, 2016). However, our sample size may not have been sufficient to detect statistically significant sex differences in EDAD. Therefore, future research with adequate sample sizes to further examine potential sex differences is needed. Second, we did not have measures of discrimination to further probe the critical association of reports of discrimination with vascular responding. Future research that includes potential moderators of macrovascular function, such as discrimination, chronic stress, and depression, is needed. Third, our study did not include a measure of systemic sympathetic nervous system activity, such as overnight urinary norepinephrine. Future studies are needed to further clarify the role of sympathetic nervous system activity in the ethnic differences in vascular function. A Genome‐Wide Association Study in AAs identified multiple SNPs associated with SBP. Two of these genes are associated with a sodium/potassium/calcium exchanger and may be associated with BP regulation (Adeyemo et al., 2009). Future studies investigating how these SNPs are associated with hemodynamics, such as TPR and indices of vascular function (e.g., EDAD), might be warranted. Finally, as this study was cross‐sectional it is not possible to draw any causal inferences from these data. Future prospective studies are needed to explicate the causal pathways involved in the vascular function differences observed in the present study.

In summary, the present study in normotensive, emerging adults with a confirmed family history of CVD showed that AAs had higher TPR and HRV and lower EDAD compared to their EA counterparts. This was despite no difference in PP or SV. This study contributes to a growing body of research showing important differences in vascular function between AAs and EAs. Future studies are needed to further characterize these differences and provide insights into potential interventions to alleviate the persistent health disparity in cardiovascular morbidity and mortality.

## AUTHOR CONTRIBUTIONS

G.K.K. conceived and designed research and performed experiments. J.F.T. and D.K.W. analyzed data, interpreted results of experiments. D.K.W. prepared figures and tables. J.F.T., D.K.W., and J.B. drafted and revised manuscript. J.F.T., D.K.W., D.P.W., J.K., and M.J. edited manuscript, G.K.K. and J.F.T. approved final version of manuscript.

## FUNDING INFORMATION

No funding information provided.

## CONFLICT OF INTEREST STATEMENT

No conflicts of interest, financial or otherwise, are declared by the authors.

## ETHICS STATEMENT

The Institutional Review Board at the study site approved all procedures and the study was performed according to the ethical standards of the 1964 Declaration of Helsinki and The Code of Ethics of the World Medical Association. Informed consent was obtained from all participants.

## Supporting information


Table S1.

## Data Availability

Analyses and generated data sets that support the current study are not available publicly. The data sets are available from the corresponding author on reasonable request.
