# Supplementary material for: African Americans with a family history of cardiovascular disease show lower endothelial‐dependent vasodilation
Source: Physiol Rep. 2025 Mar 28;13(7):e70176. doi: 10.14814/phy2.70176 (PMC11950637; doi:10.14814/phy2.70176)
Supplement: Supplementary file 1 — Table S1. [file PHY2-13-e70176-s001.docx]

Supplemental Table S1. Descriptive statistics by sex

|  | **A: Full Sample** | | **B: Women** | | **C: Men** | |  |  |  |  |
| --- | --- | --- | --- | --- | --- | --- | --- | --- | --- | --- |
|  | **Mean** | **SD** | **Mean** | ***SD*** | **Mean** | ***SD*** | ***t / X2*** | ***r*** | ***95%CI*** | ***p*** |
| Age | 23.15 | 2.82 | 23.39 | 2.74 | 22.86 | 2.90 | 1.61 | .15 | 0.03, 0.26 | **.109** |
| BMI (kg/m2) | 28.42 | 7.97 | 29.63 | 8.73 | 26.93 | 6.65 | 2.94 | .16 | 0.05, 0.27 | **.004** |
| Education (years) | 13.49 | 2.36 | 13.47 | 2.10 | 13.52 | 2.66 | -0.18 | .24 | 0.12, 0.34 | .858 |
| Sex (Women/Men) | 164/132 | | 164 | | 132 | | 5.50 | |  | **.019** |
| HF-HRV (ms2) | 7.21 | 1.03 | 7.20 | 1.09 | 7.23 | 0.95 | 0.19 | .01 | -0.10, 0.13 | .847 |
| HR (bpm) | 64.05 | 9.05 | 66.43 | 9.38 | 61.11 | 7.71 | 4.52 | .26 | 0.14, 0.37 | **<.001** |
| SV (mL/beat) | 80.87 | 19.26 | 74.42 | 16.82 | 88.83 | 19.17 | 7.95 | .42 | 0.31, 0.54 | **<.001** |
| Cardiac Output (L/min) | 5.14 | 1.29 | 4.93 | 1.27 | 5.41 | 1.28 | 4.53 | .26 | 0.14, 0.37 | **<.001** |
| Cardiac Index (L/min) | 2.65 | 0.57 | 2.66 | 0.59 | 2.65 | 0.54 | 0.35 | .02 | -0.09, 0.14 | .724 |
| TPR (mmHg/l/min) | 16.79 | 4.57 | 17.30 | 4.78 | 16.15 | 4.24 | 2.87 | .17 | 0.05, 0.28 | **.004** |
| TPI (mmHg/L/min/m2) | 31.96 | 7.75 | 31.50 | 7.69 | 32.53 | 7.81 | 1.78 | .10 | -0.01, 0.22 | .076 |
| MAP (mmHg) | 81.06 | 8.22 | 79.89 | 8.85 | 82.51 | 7.13 | 4.15 | .24 | 0.12, 0.35 | **<.001** |
| SBP (mmHg) | 112.92 | 11.85 | 109.33 | 11.23 | 117.35 | 11.11 | 7.78 | .41 | 0.30, 0.53 | **<.001** |
| DBP (mmHg) | 63.03 | 7.77 | 63.99 | 8.26 | 61.85 | 6.97 | 1.99 | .12 | 0.00, 0.23 | **.048** |
| Pulse Pressure (mmHg) | 49.89 | 10.50 | 45.34 | 8.38 | 55.50 | 10.17 | 11.02 | .54 | 0.43, 0.66 | **<.001** |
| EDAD (%) | 12.40 | 7.15 | 12.82 | 7.24 | 11.88 | 7.02 | 1.22 | .07 | -0.04, 0.19 | .222 |

Note: This table presents the means and standard deviations of variables of interest in the full sample (A) and stratified by women (B) and men (C). Pearson Chi-Square for differences between women and men (X^2^), Means (M), standard deviations (SD), t-values (t), effect size (r), associated 95% confidence intervals for r’s (95% CI) and Analysis of Variance t- and p-value statistics for the difference between women and men. Age in years, body mass index (BMI) measured in kg/m2; father’s education measured in years; HR (heart rate) in beats per minute; high-frequency power heart rate variability (HF-HRV) in milliseconds squared (ms2); stroke volume (SV) in milliliters per beat (ml/beat); cardiac output in liters per minute (l/min); adjusted CO (cardiac index; CI) in liters per min per meters squared (l/min/m^2^); total peripheral resistance (TPR) in millimeters of mercury per liter per minute (mmHg/l/min); body surface area adjusted TPR (TPI) in millimeters of mercury per liter per meters squared (mmHg/l/min/m^2^); mean arterial pressure (MAP) in millimeters of mercury (mmHg); systolic blood pressure (SBP) in millimeters of mercury (mmHg); diastolic blood pressure (SBP) in millimeters of mercury (mmHg); pulse pressure in millimeters of mercury (mmHg); and endothelial-dependent arterial dilation (EDAD) expressed as a percent (%). Significant p-values are bolded (*p <* .05).
